# Immunity-related genes in *Ixodes scapularis*—perspectives from genome information

**DOI:** 10.3389/fcimb.2014.00116

**Published:** 2014-08-22

**Authors:** Alexis A. Smith, Utpal Pal

**Affiliations:** Department of Veterinary Medicine, Virginia-Maryland Regional College of Veterinary Medicine, University of MarylandCollege Park, MD, USA

**Keywords:** ticks, *Ixodes scapularis*, *Borrelia burgdorferi*, immunity-related genes, innate response, genomics

## Abstract

*Ixodes scapularis*, commonly known as the deer tick, transmits a wide array of human and animal pathogens including *Borrelia burgdorferi*. Despite substantial advances in our understanding of immunity in model arthropods, including other disease vectors, precisely how *I. scapularis* immunity functions and influences persistence of invading pathogens remains largely unknown. This review provides a comprehensive analysis of the recently sequenced *I. scapularis* genome for the occurrence of immune-related genes and related pathways. We will also discuss the potential influence of immunity-related genes on the persistence of tick-borne pathogens with an emphasis on the Lyme disease pathogen *B. burgdorferi.* Further enhancement of our knowledge of tick immune responses is critical to understanding the molecular basis of the persistence of tick-borne pathogens and development of novel interventions against the relevant infections.

## Introduction

Although several hundred tick species are known to exist (Jongejan and Uilenberg, 2004), only a handful transmit human diseases. *Ixodes scapularis* is one of the predominant tick species that spread a wide array of serious human and animal pathogens, including *Borrelia burgdorferi*, which causes Lyme borreliosis (Burgdorfer et al., 1982; Anderson, 1991). Our understanding of arthropod innate immune responses, primarily involving the fruit fly and mosquito, has advanced over the past decades (Vilmos and Kurucz, 1998). However, our knowledge of tick immune responses, especially the occurrence of immune-related genes, pathways, and specifically how these components respond to invading pathogens remains under-explored. Notably, many pathogens that persist in and transmit through ticks are evolutionarily distinct and possess unique structures (Hajdusek et al., 2013). For example, key pattern recognition molecules (PAMPs), such as peptidoglycan (PG) and lipopolysaccharides (LPS), are structurally different or completely absent, respectively, in major tick-borne pathogens, such as in *B. burgdorferi* (Schleifer and Kandler, 1972; Takayama et al., 1987; Fraser et al., 1997). Thus, the wealth of knowledge generated in other model arthropods, especially regarding the genesis of host immune responses against classical Gram-positive or Gram-negative bacterial pathogens, might not be readily applicable for tick-borne pathogens, like *B. burgdorferi.* The primary goal of this review is to present a general overview of tick immune components, as gathered from the sequenced genome and published data, and discuss their potential for modulating infection, with a focus on a major tick-borne pathogen, *B. burgdorferi*. A better understanding of the *I. scapularis* immune response to invading pathogens could contribute to the development of new strategies that interfere with relevant pathogen persistence and transmission.

While a number of studies detailed characterization of *I. scapularis* proteins, predominantly salivary gland proteins, that influence immunity and pathogen persistence in the vertebrate hosts (Wikel, 1996; Das et al., 2001; Gillespie et al., 2001; Narasimhan et al., 2002, 2004, 2007; Hovius et al., 2008; Dai et al., 2010; Pal and Fikrig, 2010; Kung et al., 2013), relatively limited information is available on how tick proteins shape vector immunity and influence pathogen persistence. In order to generate a list of tick immune genes and related pathways, we sought to perform a comprehensive analysis of the recently sequenced *I. scapularis* genome data that are available through several publicly accessible databases (Hill and Wikel, 2005; Pagel Van Zee et al., 2007). To accomplish this, we initially searched the National Institute of Allergy and Infectious Diseases Bioinformatics Resource Center (www.vectorbase.org) for annotated *I. scapularis* immune-related genes. In addition, we also reviewed the relevant literature to identify additional innate immune genes, including those discovered in related tick species (Rudenko et al., 2005) or in fruit fly, mosquito, and mammalian genomes (Sonenshine, 1993; Hoffmann et al., 1999; Dimopoulos et al., 2000; Christophides et al., 2002; Hoffmann and Reichhart, 2002; Janeway and Medzhitov, 2002; Govind and Nehm, 2004; Osta et al., 2004; Saul, 2004; Tanji and Ip, 2005; Dong et al., 2006; Ferrandon et al., 2007; Tanji et al., 2007; Jaworski et al., 2010; Kopacek et al., 2010; Yassine and Osta, 2010; Valanne et al., 2011). The latter information was then used to search for possible *Ixodes* orthologs via BLASTP against the VectorBase database. In total, 234 genes were identified and categorized into one of the following nine major immune pathways or components (number of unique genes): gut-microbe homeostasis (17), agglutination (37), leucine-rich repeat (LRR) proteins (21), proteases (33), coagulation (11), non-self recognition and signal transduction via Toll, IMD, and JAK-STAT pathways (55), free radical defense (13), phagocytosis (33), and anti-microbial peptides (14). These genes are listed in Tables 1–9; unless stated otherwise, all annotations are based on the VectorBase database. We recognize that although our list might not be comprehensive as there might be additional published data inadvertently overlooked in our literature/database searches or yet-to-be identified genes involved in tick immune defense, we believe that it still represents the majority of genes that are potentially involved in the tick immune response. In the following sections, occurrence of these components and pathways are systematically discussed for their occurrence in the tick genome; we also highlighted their potential influence on the persistence and transmission of tick-borne pathogens like *B. burgdorferi*.

**Table 1:** **Gut-microbe homeostasis**.

**Annotation**	**Accession number**
Dual oxidase	ISCW007865
Phospholipid-hydroperoxide glutathione peroxidase, putative	ISCW019584
Phospholipid-hydroperoxide glutathione peroxidase, putative	ISCW022517
Glutathione peroxidase, putative	ISCW020571
Peroxidase	ISCW017070
Peroxidase	ISCW022537
Glutathione peroxidase, putative	ISCW008495
Oxidase/peroxidase, putative	ISCW002528
Phospholipid-hydroperoxide glutathione peroxidase, putative	ISCW015098
Oxidase/peroxidase, putative	ISCW017368
Oxidase/peroxidase, putative	ISCW005828
Peroxidase	ISCW024650
Glutathione peroxidase, putative	ISCW020569
Oxidase/peroxidase, putative	ISCW018825
Peroxidase	ISCW013159
Thioredoxin peroxidase, putative	ISCW013767
Glutathione peroxidase, putative	ISCW001759

**Table 2:** **Agglutination**.

**Annotation**	**Accession number**
Ferritin	ISCW015079
Beta-galactosidase	ISCW000651
Ubiquitin associated domain containing protein	ISCW023764
Chitin bindin peritrophin A	ISCW006076
Beta-galactosidase precursor	ISCWOI9676
galectin, putative	ISCW008553
Manose binding ER-golgi comparment lectin	ISCW016179
Ixoderin precursor	ISCW002664
Ixoderin precursor	ISCW022063
Ixoderin B	ISCW013797
Ixoderin B	ISCW003711
Hemelipoglycoprotein precursor	ISCW012423
Ferritin	ISCW023334
galectin, putative	ISCW020268
Beta-galactosidase	ISCW019681
Beta-galactosidase precursor	ISCW019677
Beta-galactosidase precursor	ISCW019679
Beta-galactosidase precursor	ISCW0l6637
Hemelipoglycoprotein precursor	ISCW024299
fatty acyl-CoA elongase, putative	ISCW010899
Galectin	ISCW020268
Ixoderin Precursor	ISCW024686
Hemelipoglycoprotein precursor	ISCW0l2424
Sodium/proton exchanger	ISCW008652
C-Type Lectin, Putative	ISCW010467
Ixoderin Precursor	ISCW013746
Double sized immunoglobulin g binding protein A	ISCW021766
Galectin	ISCW008553
Hemelipoglycoprotein precursor	ISCW021704
Beta-galactosidase precursor	ISCW019678
Lectin, Putative	ISCW012623
Galectin	ISCW020586
Hemelipoglycoprotein precursor	ISCW014675
Hemelipoglycoprotein precursor	ISCW021709
Ixoderin precursor	ISCW012248
Ubiquitin associated and SH3 domain containing protein B	ISCW02l035
Galectin, putative	ISCW020586

**Table 3:** **Leucine-rich repeat (LRR) proteins**.

**Annotation**	**Accession number**
Lumicans	ISCW001027
LRR (in flii) interacting protein	ISCW016609
Lumicans	ISCW005645
F-Box/LRR protein, putative	ISCW000110
F-Box/LRR protein, putative	ISCW010598
F-Box/LRR protein, putative	ISCW010597
F-Box/LRR protein, putative	ISCW010599
LRR protein, putative	ISCW008095
LRR protein	ISCW014626
F-Box/LRR protein, putative	ISCW010347
F-Box/LRR protein, putative	ISCW005273
LRR and NACHT domain containing protein	ISCW004678
LRR and NACHT domain containing protein	ISCW001292
LRR protein	ISCW012038
F-Box/LRR protein, putative	ISCW016452
F-Box/LRR protein, putative	ISCW018961
F-Box/LRR protein, putative	ISCW013925
LRR containing G-protein coupled receptor	ISCW015788
F-Box/LRR protein, putative	ISCW018134
F-Box/LRR protein, putative	ISCW008236
LRR protein, putative	ISCW003174

**Table 4:** **Proteases/Protease inhibitors**.

**Annotation**	**Accession number**
Serpin 4 precursor	ISCW023622
Serpin 2 precursor	ISCW010422
Serpin 2 precursor	ISCW018607
Serpin 1 precursor	ISCW023618
Serpin 7 precursor	ISCW024109
PAP associated domain containing protein	ISCW014870
Serpin 7 precursor	ISCW009616
Serine proteinase inhibitor serpin-3	ISCW015204
Heparan sulfate 2-O sulfotransferase, putative	ISCW000208
Secreted salivary gland peptide	ISCW023621
Serpin	ISCW016489
Hypothetical protein	ISCW017929
Secreted salivary gland peptide	ISCW023620
Serpin 4 precursor	ISCW023623
Protein disulfide isomerase 1	ISCW002080
Alkaline phosphatase	ISCW023785
Alkaline phosphatase	ISCW003801
Alkaline phosphatase	ISCW004677
Hypothetical protein	ISCW021544
Zinc metalloprotease	ISCW008637
Zinc metalloprotease	ISCW005798
Zinc metalloprotease	ISCW005687
Zinc metalloprotease	ISCW005854
Serpin 8 precursor	ISCW014652
Serpin	ISCW014100
Zinc metalloprotease	ISCW012815
Conserved hypothetical protein	ISCW006169
Serpin 8 precursor	ISCW015349
Zinc metalloprotease	ISCW021286
Serpin 2 precursor	ISCW021417
Serpin 2 precursor	ISCW014779
Secreted serine protease	ISCW014551
Alkaline phosphatase	ISCW000162

**Table 5:** **Coagulation**.

**Annotation**	**Accession number**
Proclotting enzyme precursor	ISCW013112
Thrombin inhibitor	ISCW000427
Proclotting enzyme precursor	ISCW000320
Proclotting enzyme precursor	ISCW011206
Proclotting enzyme precursor	ISCW001322
Keratinocyte transglutaminase	ISCW019475
Proclotting enzyme precursor	ISCW011961
Proclotting enzyme precursor	ISCW003779
Proclotting enzyme precursor	ISCW010999
Prostate-specific transglutaminase	ISCW009303
Prostate-specific transglutaminase	ISCW011739

**Table 6:** **Non-self recognition (Toll, IMD, and JAK-STAT pathways)**.

**Annotation**	**Accession number**
Regulator of ubiqutin pathway, putative	ISCW015648
NF-kappaB inhibitor IkappaB, putative	ISCW007030
Peptidoglycan recognition receptor protein	ISCW022212
Embryonic polarity dorsal, putative	ISCW000140
Ankyrin repeat-containing protein	ISCW018861
Peptidoglycan recognition receptor protein	ISCW024175
Netrin receptor DSCAM	ISCW016844
Netrin receptor DSCAM	ISCW016100
Caspase, apoptotic cysteine protease, putative	ISCW003039
Netrin receptor DSCAM	ISCW020406
Peptidoglycan recognition receptor protein	ISCW004389
Netrin receptor DSCAM	ISCW022828
Scavenger receptor class B	ISCW003371
Scavenger receptor class B	ISCW010934
Scavenger receptor class B	ISCW002412
Netrin receptor DSCAM	ISCW003295
UBX domain-containing protein, putative	ISCW011870
NF-kappaB inhibitor IkappaB, putative	ISCW019520
Nuclear factor nf-kappa-B P105 subunit, putative	ISCW018935
Peptidoglycan recognition receptor protein	ISCW024689
N-CAM Ig domain containing protein	ISCW022144
Toll	ISCW018193
Secreted protein, putative	ISCW021005
Secreted protein, putative	ISCW024521
Toll	ISCW018363
Serine-threonine protein kinase, plant-type, putative	ISCW001463
Toll	ISCW004495
Toll	ISCW020989
Spatzle	ISCW022569
Fibrinogen	ISCW009412
Tartan protein, putative	ISCW016292
Secreted protein, putative	ISCW024389
Toll	ISCW007724
Toll	ISCW007726
Myd88, putative	ISCW008802
Fibrinogen	ISCW009412
Toll	ISCW009512
Toll	ISCW022740
Serine/threonine protein kinase	ISCW020049
Toll	ISCW017724
Tartan protein, putative	ISCW021508
Kekkon 1, putative	ISCW018006
Toll	ISCW020221
Tolkin	ISCW022120
Membrane glycoprotein LIG-1, putative	ISCW005558
Slit Protein	ISCW018651
Toll	ISCW008289
Adenylate cyclase	ISCW012040
Toll	ISCW006897
Fibrinogen	ISCW024309
Toll	ISCW007727
Spatzle	ISCW022732
Fibrinogen	ISCW001478
JAK	ISCW016158
STAT 3	ISCW005692

**Table 7:** **Free radical defense**.

**Annotation**	**Accession number**
Manganese superoxide dismutase	ISCW016585
Superoxide dismutase	ISCW015027
Manganese superoxide dismutase	ISCW016737
Superoxide dismutase Cu-Zn	ISCW012382
Nitric oxide synthase interacting protein, putative	ISCW017590
Superoxide dismutase	ISCW018077
Superoxide dismutase	ISCW024422
Cu^2+^/Zn^2+^ superoxide dismutase SODI	ISCW011852
Manganese superoxide dismutase	ISCW012767
Superoxide dismutase Cu-Zn	ISCW008219
Ras responsive element binding protein	ISCW009132
Decarboxylase	ISCW021675
Nitric oxide synthase	ISCW018074

**Table 8:** **Phagocytosis**.

**Annotation**	**Accession number**
Integrin beta-3	ISCW024103
Cadherin- repeats domain containing protein	ISCW013741
Rho GTPase activating protein	ISCW015851
Protocadherin fat	ISCW017319
Thioester containing protein	ISCW020822
Cadherin	ISCW005817
Rho GTPase activating protein	ISCW015201
Protocadherin beta-6	ISCW016805
Integrin alpha-ps	ISCW005672
GTPase Rho	ISCW004349
Rho guanine nucleotide exchange factor	ISCW014238
GTPase Rho	ISCW004348
Protocadherin fat	ISCW016765
Rho GTPase activating protein RICH2	ISCW003282
Integrin alpha	ISCW003186
Integrin beta-3	ISCW010037
GTPase Rho	ISCW006741
Rho GTPase activating protein	ISCW003559
Integrin beta subunit	ISCW008948
p116 Rho-interacting protein	ISCW007121
Integrin alpha repeat domain containing protein	ISCW019648
Rho GTPase activating protein	ISCW018998
Rho guanine exchange factor	ISCW008875
Rho	ISCW002009
Integrin alpha-ps	ISCW022321
Rho GTPase activating protein	ISCW019271
GTPase Rho	ISCW018929
Rho GTPase activating protein	ISCW001560
Rho GDP dissociation inhibitor	ISCW020878
Rho associated kinase	ISCW011682
GTPase Rho	ISCW015794
Integrin beta subunit	ISCW002553
Integrin alpha	ISCW003185

**Table 9:** **Antimicrobial peptides**.

**Annotation**	**Accession number**
Putative secreted salivary gland peptide	ISCW005928
Secreted protein, putative	ISCW018425
Secreted salivary gland peptide	ISCW002695
TAK 1 putative	ISCW009364
Secreted salivary gland peptide	ISCW001310
Beta transducin Trp-Asp domain containing protein	ISCW014204
Map kinase activating death domain protein	ISCW017494
Secreted salivary gland peptide	ISCW018541
Defensin	ISCW022102
Preprodefensin putative	ISCW016747
Secreted salivary gland peptide	ISCW002331
Secreted salivary gland peptide	ISCW016466
Secreted salivary gland peptide	AAV63544^*^
Arsenite-resistance protein	ISCW011320

* Based on annotation in NCBI database (http://www.ncbi.nlm.nih.gov).

## *I. scapularis* genome

The *I. scapularis* genome is relatively large, approximately 2.1 Gb in size and contains nearly 70% repetitive DNA (Ullmann et al., 2005). Recently it was completely sequenced by the *I. scapularis* genome project - a partnership between a number of tick research communities and institutions (Hill and Wikel, 2005; Pagel Van Zee et al., 2007). Toward the end of 2008, sequencing centers announced the annotation and release of the whole genome sequence data (IscaW1, 2008; GenBank accession ABJB010000000). The sequence data were derived from purified genomic DNA preparations isolated from an in-bred tick colony and sequenced to approximately 6-fold coverage using a combined whole genome shotgun and clone-based approach. The genome information are organized and displayed by a bioinformatics resource center focused on invertebrate vectors of human disease called VectorBase (www.vectorbase.org), which is funded by the National Institute of Allergy and Infectious Diseases, National Institutes of Health. The *I. scapularis* gene counts included 20,486 high confidence protein-coding genes, 316 non-coding genes and 20,771 transcripts. While the most recent release (IscaW1.3; 2014) reported no modifications of protein-coding loci, it incorporated a new prediction for 285 non-coding RNAs.

## Immunity-related gene/pathways in *I. scapularis*

### Gut microbe homeostasis

Gut microbiota serve a critically important function in shaping host immunity in a number of organisms, including model arthropods (Dillon and Dillon, 2004; Round and Mazmanian, 2009; Hooper et al., 2012; Buchon et al., 2013; Kamada et al., 2013; Schuijt et al., 2013). Characterization of gut microbiota in ticks, including *I. scapularis*, as well as their influence on the persistence of tick-borne pathogens like *B. burgdorferi* has been a focus of a number of recent studies (Clay et al., 2008; Carpi et al., 2011; Narasimhan et al., 2014). As many of these gut microbes play a beneficial role in the physiology of the host, the immune system therefore must be able to differentiate between commensal microbes and pathogenic microorganisms (Macpherson and Harris, 2004). While mechanisms that contribute to the microbial surveillance and pathogen elimination while tolerating the indigenous microbiota remain obscure in ticks, these are well-researched in many arthropods, particularly in *D. melanogaster* (Buchon et al., 2013). Studies have established that immune reactivity within the fly gut ensures preservation of beneficial and dietary microorganisms, while mounting robust immune responses to eradicate pathogens (Buchon et al., 2013). There are at least two models of fly immunity for sensing and preserving beneficial bacterial associations while eliminating potentially damaging ones (Lazzaro and Rolff, 2011). The first occurs by recognition of non-self molecules (invading microbes), while the second involves the recognition of “danger” signals that are released by damaged host cells. However, it is also likely that they work together to maintain effective gut microbe homeostasis. Recent studies suggest that dual oxidase (DUOX) and peroxidases enzymes play a key role in this process (Kim and Lee, 2014). While a number of other regulatory molecules may participate in gut homeostasis, we classified 17 different genes within the *I. scapularis* genome to this pathway, including a single dual oxidase (DUOX) and several peroxidase proteins (Table 1).

Additional studies have recently detailed how DUOX plays an essential role in gut mucosal immunity and homeostasis (Bae et al., 2010; Deken et al., 2013). DUOX, a member of the nicotinamide adenine dinucleotide phosphate (NADPH) oxidase NOX family (Geiszt and Leto, 2004), has previously been shown to be a key source of local microbicidal reactive oxygen species (ROS) production within the fly gut (Kim and Lee, 2014). Targeted depletion of DUOX in flies has resulted in the overproduction of commensal gut bacteria and renders the flies susceptible to infection (Buchon et al., 2013; Kim and Lee, 2014). As originally discovered in *Caenorhabditis elegans* (Edens et al., 2001), in addition to ROS generation, DUOX is also implicated for catalysis of protein cross-linking that contributes to maintenance of gut microbiota in *Anopheles gambiae* (Kumar et al., 2010). In mosquitoes, DUOX, along with a specific heme-peroxidase, catalyzes the formation of an acellular molecular barrier, termed dityrosine network (DTN), which forms in the luminal space along the gut epithelial layer during feeding (Kumar et al., 2010). The DTN decreases the gut permeability to various immune elicitors protecting the gut microbiota, both commensal and pathogenic species. Another recent study revealed that an ovarian dual oxidase is essential for insect eggshell hardening through the production of H_2_O_2_,which ultimately promotes protein cross-linking (Dias et al., 2013). Further studies on how DUOX and peroxidase systems maintain gut microbiota in *I. scapularis* could give novel insight into how pathogens that are transmitted through ticks are able to evade the immune system and persist within the vector.

### Agglutination

Agglutination, the biological phenomenon by which cells or particles clump together, has been described within various tick species (Uhlir et al., 1996; Kibuka-Sebitosi, 2006). A group of carbohydrate-binding proteins called lectins (Grubhoffer et al., 1997, 2004), which are often produced in a tissue specific manner within arthropods, especially in the gut, hemocytes, or fat bodies, could be key mediators of the process (Grubhoffer et al., 2004, 2005). Agglutination of pathogens by lectins, which also function as host recognition receptors for pathogen-associated molecular patterns (Dam and Brewer, 2010), has been reported in many arthropod vectors, including mosquitoes and tsetse flies, where they play an important role in the pathogen-host relationship (Abubakar et al., 1995, 2006; Barreau et al., 1995; James, 2003). While lectins can function as signaling factors for the maturation of the African trypanosome or as lytic factors (Abubakar et al., 1995, 2006), in mosquitoes they act as agonists of the development of malarial parasites within the vector (Barreau et al., 1995; James, 2003) While tick lectins, particularly those in hard ticks (*Ixodidae*), have not been studied as extensively as other arthropod lectins, previous reviews summarized available information on lectins of *I. ricinus* (Grubhoffer and Jindrak, 1998; Grubhoffer et al., 2004). Since most lectins isolated from arthropods are the ones from the hemocoel, studies have focused on their localization or hemagglutinating activity in the hemolymph (Sonenshine, 1993; Kuhn et al., 1996). In *I. ricinus*, this activity was characterized as Ca^2+^ dependent binding activity (Grubhoffer et al., 2004). A 85 kDa lectin produced by the granular hemocytes and basal laminae surrounding the hemocoel was identified to have a strong binding affinity for sialic acid (Grubhoffer et al., 2004). This immunoreactivity supports the idea that lectins may function as a recognition molecule of the immune system in ticks, implying that they could influence the persistence of tick-borne pathogens like *B. burgdorferi*. In fact, the hemocytes in *I. ricinus* can also phagocytize *B. burgdorferi* through the coiling method, which has previously been though to be a lectin-mediated process (Grubhoffer and Jindrak, 1998). Specifically, two agglutinins/lectins were isolated from the gut, one 65 kDa and the other 37 kDa in size; the former was shown to be the main agglutinin with a binding affinity for mucin, while the latter protein was found to have a strong affinity for a specific glucan (Grubhoffer and Jindrak, 1998; Grubhoffer et al., 2004). It is also suggested that a gut agglutinin has the potential to bind LPS that in cooperation with other digestive enzymes thought to affect the persistence of Gram-negative bacteria and spirochetes that pass through the gut lumen (Uhlir et al., 1996; Grubhoffer et al., 2004). In addition to hemolymph and gut, lectin activities are also documented in the salivary gland; a 70 kDa protein has been identified as being responsible for the hemagglutinating activity in this organ (Grubhoffer et al., 2004). It is thus possible that lectin or a related protein in the salivary glands could influence pathogen transmission. In fact, a tick mannose-binding lectin inhibitor that is produced in the salivary glands has been shown to interfere with the human lectin complement cascade, significantly impacting the transmission and survival of *B. burgdorferi* (Schuijt et al., 2011). Taken together, it is likely that lectins could play a role in the immunity of *I. scapularis*, which encodes for at least 37 lectins or related proteins (Table 2).

### Leucine-rich repeat proteins

LRR have previously been shown to occur in more than 2000 proteins throughout the plant and animal kingdom, including Toll-like receptors, and are thought to play an essential role in host defense (Boman and Hultmark, 1981; Kobe and Kajava, 2001; Bell et al., 2003; Enkhbayar et al., 2004). LRR proteins typically contain 20–29 amino acid residues (with repeats ranging from 2 to 42) that are involved in protein-protein interactions with diverse cellular locations and functions. While the biological significance of LRR containing proteins in ticks remains unknown, notably, the *I. scapularis* genome encodes at least 22 potential LRR proteins (Table 3). Unlike in ticks, the roles of LRR proteins in the immunity of other arthropods, including blood-meal seeking arthropods, however, are relatively well-characterized (Povelones et al., 2009, 2011). For example, in *Anopheles gambiae*, LRR-containing proteins, such as LRIM1 and APL1C, have been identified as a potent antagonist of malarial parasites, limiting *Plasmodium* infection by activating a complement-like system (Fraiture et al., 2009; Povelones et al., 2009, 2011; Baxter et al., 2010). In *Manduca sexta*, an LRR-containing protein, termed leureptin, is shown to bind lipopolysaccharide and is involved in hemocyte responses to bacterial infection (Zhu et al., 2010). Further studies into how tick LRR-containing proteins contribute to vector immunity and influence pathogen persistence are warranted.

### Proteases/protease inhibitors

A number of immune cascades that serve to recognize and control invading pathogens are dependent on the activity of specific proteases or protease inhibitors (Janeway and Medzhitov, 2002; Sojka et al., 2011). Proteases, specifically serine proteases, have previously been shown to be a key regulating molecule for several of these immune response pathways, including coagulation, antimicrobial peptide synthesis, and melanization of pathogens (Gorman and Paskewitz, 2001; Janeway and Medzhitov, 2002; Jiravanichpaisal et al., 2006). Such serine protease-dependent cellular response, for example, as demonstrated for coagulation in the horseshoe crab, manifests through the rapid activation of immune pathways in response to pathogen detection (Hoffmann et al., 1999; Fujita, 2002). Activation of this pathway has been shown to be controlled by three serine proteases: factor C, factor B, and a pro-clotting enzyme (Tokunaga et al., 1987). When LPS is present, clotting factors that are stored within hemocytes are readily released into the hemolymph, which ultimately results in the immobilization of the invading pathogen.

Protease inhibitors also control a variety of proteolytic pathways and are known to play an important role in arthropod immunity (Kanost, 1999). A group of serine protease inhibitors, termed serpins, have been the focus of many recent studies that demonstrate the critical contribution of these proteins to the regulation of inflammation, blood coagulation, and complement activation in mammals (Kanost, 1999). Serpins are also shown to contribute to immunity and physiology in arthropods, as shown in mosquitoes (Gulley et al., 2013) and flies (Reichhart et al., 2011). A detailed characterization of serpins in ticks, including *I. scapularis*, has been reported by Mulenga et al. (Mulenga et al., 2009). These authors reported the presence of at least 45 serpin genes within the *I. scapularis* genome, interestingly, most of which are differentially expressed in the gut and salivary glands of unfed and partially fed ticks (Mulenga et al., 2009). It is speculated that ticks could utilize some of these serpins to manipulate host defense to facilitate tick feeding and subsequent disease transmission, although the precise role of serpins in the physiology and immunity within the tick vector awaits further investigation. More recently, a novel serpin, termed IRS-2, was described in *I. ricinus* (Chmelar et al., 2011). IRS-2 was shown to inhibit cathepsin G and chymase, thereby inhibiting host inflammation and platelet aggregation. This particular protein was also thought to act as a modulator of vascular permeability. Although whether serpins play a role in host microbe interactions remains unknown, studies also explored their potential as target antigens for development of a tick vaccine (Muleng et al., 2001).

### Coagulation

Injury as well as the presence of microbes in arthropods could result in the induction of two major proteolytic pathways - coagulation and melanization (Theopold et al., 2004). Key enzymes for these processes that cross-link the clot or induce a proteolytic pathway similar to the vertebrate clotting cascade include transglutaminase and phenoloxidase, respectively. Studies in the horseshoe crab have provided a breakthrough in our understanding of the coagulation pathway in arthropods (Theopold et al., 2004). This pathway is characterized by a rapid sequence of highly localized serine proteases and culminates in the generation of thrombin; the process is tightly regulated to ensure excessive clot formation does not occur (Crawley et al., 2011). The *I. scapularis* genome encodes for at least 11 genes that may be part of the coagulation pathway (Table 5), although precisely how this pathway controls wound healing or affects microbial survival remains unknown. Notably, while the *I. scapularis* genome lacks genes related to the melanization (phenoloxidase) pathway, phenol oxidase activity was detected in the hemolymph of the soft ticks, *Ornithodoros moubata* (Kadota et al., 2002).

### Non-self recognition and signal transduction pathways (Toll, IMD, and JAK-STAT)

**Figure 1:**
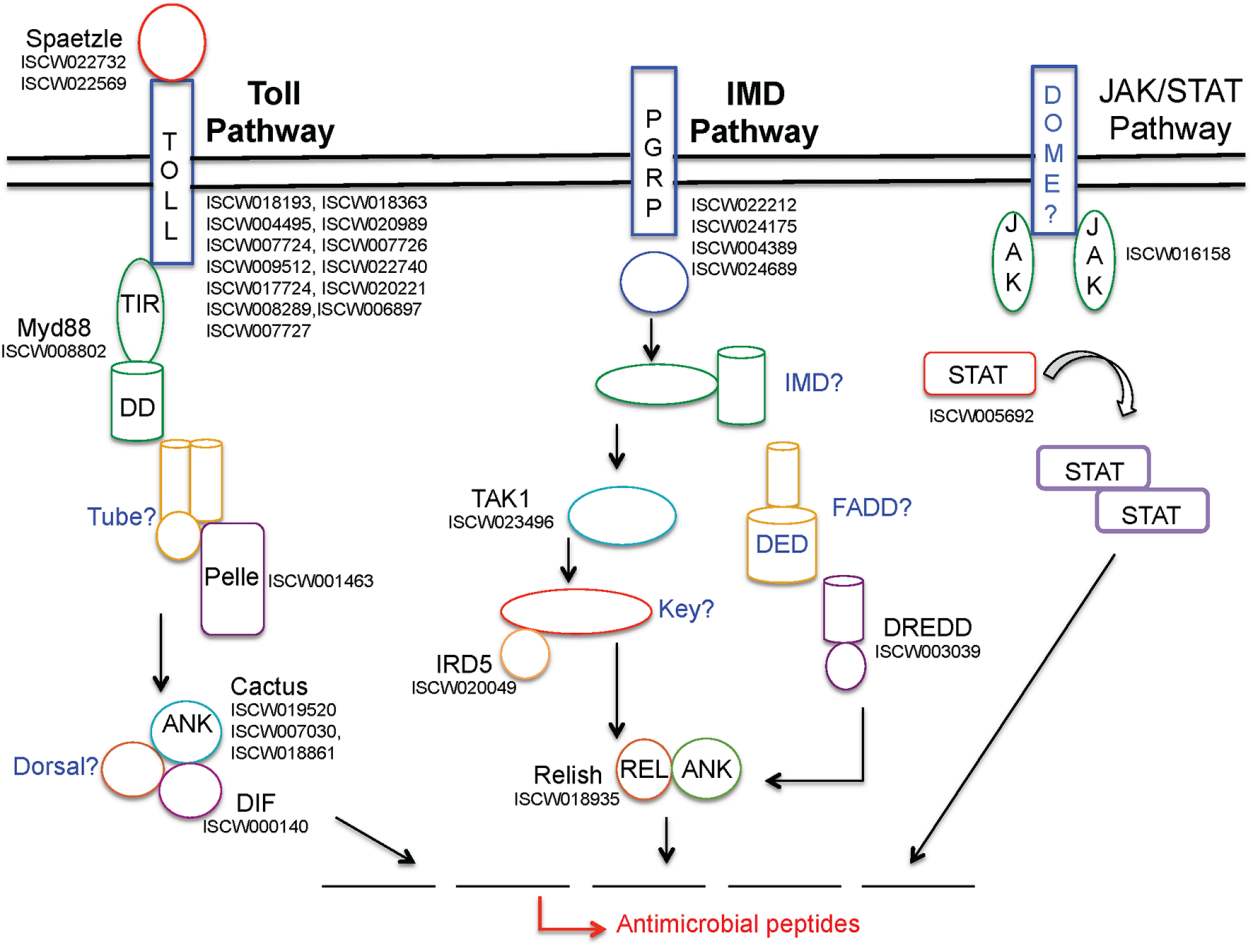
**Schematic representation of *I. scapularis* immune-related genes potentially representing three major signaling pathways that lead to anti-microbial responses**. While annotated tick genes from each pathway are shown in black font, the apparently missing genes are indicated in blue. For further details on these pathways, including abbreviations, please refer to the text and earlier publications (Lemaitre, 2004; Liu et al., 2012).

Three major pathways, namely Toll, immune deficiency (IMD), and Janus kinase (JAK)- signaling transducer and activator of transcription (STAT) pathways, contribute to the activation of the immune response within arthropods, as previously detailed (Belvin and Anderson, 1996; De Gregorio et al., 2002; Hoffmann and Reichhart, 2002; Govind and Nehm, 2004; Lemaitre, 2004; Rawlings et al., 2004; Kaneko and Silverman, 2005; Tanji and Ip, 2005; Zambon et al., 2005; Tanji et al., 2007; Xi et al., 2008; Souza-Neto et al., 2009; Valanne et al., 2011; Liu et al., 2012). Notably, the *I. scapularis* genome encodes many representative genes from all three pathways (Figure 1). While Toll pathways are activated in the presence of bacterial, viral, and fungal pathogens, the IMD pathway is induced by Gram-negative bacteria. The arthropod JAK-STAT pathway, analogous to a cytokine-signaling pathway in mammals (Shuai et al., 1993), has also previously been shown to be activated in the presence of bacterial or protozoan pathogens (Buchon et al., 2009; Gupta et al., 2009; Liu et al., 2012). The Toll pathway is most extensively studied in *Drosophila*, which encodes nine Toll receptors (Valanne et al., 2011). Cell wall components in Gram-positive bacteria stimulate this pathway, whereas the precise fungal component that induces specific Tolls is not well-defined. In both cases, stimulation of the Toll pathway causes cleavage of the protein Spätzle, which eventually leads to the activation of NF-κ B transcription factor family members Dif and Dorsal, which are homologous to mammalian c-Rel and RelA, resulting in the production of different antimicrobial peptides (AMPs) (Irving et al., 2001; Christophides et al., 2002; Hetru et al., 2003). Specifically, research in *Drosophila* has shown that Gram-positive bacteria induce the Toll pathway, leading to the generation of Toll-specific AMPs, such as drosomycin (Zhang and Zhu, 2009). While roles of Toll pathways in *I. scapularis* remain obscure, we list at least 33 genes that potentially belong to this pathway (Table 6). The IMD pathway, on the other hand, is activated by the peptidoglycan molecules present on the surface of Gram-negative bacteria that are recognized by host cells via peptidoglycan recognition receptors (PGRP) (Ferrandon et al., 2007). This recognition leads to the activation of an adaptor protein and further downstream signaling molecules, such as transcription factor Relish, a compound Rel-Ank protein homologous to mammalian p100 and p105, ultimately resulting in the production of AMPs (Matova and Anderson, 2006; Ferrandon et al., 2007). Although the tick genome encodes at least 20 potential genes from this pathway (Table 6), similar to Toll, how the IMD pathway affects Gram-negative pathogens, including *B. burgdorferi* is unknown. A critical and common aspect in the response of both pathways is the ability to induce a specific AMP to combat microbial infections through the recognition of non-self. Interestingly, it is also thought that these two pathways can work synergistically to activate the expression of the same AMP (Tanji et al., 2007).

### Free radical defense

Free radicals, such as ROS, which include superoxide radicals (O·2), hydroxyl radicals (·OH), and other compounds, are able to react with biomolecules and cause damage to DNA, proteins, and lipids, playing as critical role in cell signaling (Thannickal and Fanburg, 2000). While ROS are important in arthropod development (Owusu-Ansah and Banerjee, 2009), they are indispensible in arthropod immunity, including activation of specific immune pathways (Pereira et al., 2001; Bubici et al., 2006; Molina-Cruz et al., 2008; Morgan and Liu, 2011). For example, mosquitoes that were previously infected with *Wolbachia* bacteria were observed to produce much higher levels of ROS (Pan et al., 2012). Nitric oxide (NOS), a highly unstable free radical gas, is another component of free radical defense shown to be toxic to both parasites and pathogens (James, 1995; Wandurska-Nowak, 2004). In insects, NOS is known to be induced following parasite infection (Dimopoulos et al., 1998; Davies, 2000).

A family of superoxide dismutases (SOD) that catalyze the conversion of these free radicals to non-toxic O_2_ and less toxic hydrogen peroxide (H_2_O_2_) are responsible for destroying any free radicals generated in the hosts. Glutathione-S-transferases (GST) also detoxify stress-causing agents, including toxic oxygen free radical species (Sharma et al., 2004). The genes encoding GSTs are shown to be induced in model arthropods upon oxidative stress and microbial challenge, including in ticks infected with *B. burgdorferi* (Rudenko et al., 2005). Despite these studies, how different free radicals or SOD detoxification systems play roles in pathogen persistence or clearance within *I. scapularis*, which encodes at least 13 genes of this pathway (Table 7), remains uncharacterized.

### Phagocytosis

Cells recognize, bind, and ingest relatively large particles in phagocytosis (Walters and Papadimitriou, 1978). This process is considered a major evolutionarily conserved cellular immune response in arthropods, mostly studied in model insects (Sideri et al., 2008), and is mediated by hemocytes, also known as blood cells, which are primarily present in the hemolymph as well as infrequently exist within various organs. Phagocytosis of microbes plays a critical role in arthropod defense, as blocking of phagocytosis in *Drosophila* mutants significantly impairs the flies' ability to survive subsequent bacterial infection (Elrod-Erickson et al., 2000). Hemocytes within the hemolymph have previously been shown to phagocytize various pathogens (Inoue et al., 2001). Whereas, although solid experimental evidence of phagocytosis of *B. burgdorferi* within *I. scapularis* is lacking, certain cell lines derived from ticks have been shown to be phagocytic to spirochetes (Mattila et al., 2007). Further studies into the phagocytic pathway of *I. scapularis*, which encodes 33 potentially related genes (Table 8), would provide insight into whether or how pathogens, such as *B. burgdorferi*, are phagocytized, as well as how tick-borne pathogens are able to escape this cellular immune response. Notably, the *I. scapularis* genome encodes for five small GTPases belonging to the Rho family that in addition to other cellular functions, are shown to play central roles in phagocytosis (Etienne-Manneville and Hall, 2002; Bokoch, 2005).

### Anti-microbial peptides

The production of AMPs, a hallmark of systemic humoral immune responses, is an important aspect of host defense in arthropods (Bulet et al., 1999). At least eight different classes of AMPs have been observed in the fruit fly, *Drosophila*. These AMPs, are mainly produced by fat bodies and secreted into the hemolymph and can then be further grouped into three different families based on their intended target: Gram-negative bacteria, Gram-positive bacteria, and fungi (Imler and Bulet, 2005). In arthropods, specific AMPs are produced as a result of activation of the Toll, IMD, or JNK-STAT pathway by the presence of bacteria, fungi, or viruses.

Among effector molecules of innate immune defense, AMPs are relatively well-studied in ticks, which likely generate classical AMPs in the gut and hemocoel (Hynes et al., 2005; Saito et al., 2009). AMPs have been found to be produced in hard ticks, such as *I. scapularis* and *Dermacentor variabilis*, as well as in the soft tick *Ornithodoros moubata* (Nakajima et al., 2002; Sonenshine et al., 2002; Hynes et al., 2005; Rudenko et al., 2005; Saito et al., 2009). *I. ricinus* induces a defensin-like gene in response to *B. burgdorferi* in a tissue-specific manner that is not capable of clearing the infection (Rudenko et al., 2005). *I. scapularis* encodes for at least 14 AMPs (Table 9). The exact role of defensin or other AMPs in clearance of tick-borne pathogens remains unclear. In addition, ticks may also produce non-classical AMPs. Although gastric digestion in ticks is primarily intracellular, degradation of blood components, such as hemoglobin, could create peptides with antimicrobial activities (Sonenshine et al., 2005). Whether these fragments would protect against pathogenic bacteria has currently not been reported.

## Concluding remarks

*I. scapularis* ticks are known to transmit a diverse set of disease agents ranging from bacterial to protozoans to viruses. A number of studies explored the immunomodulatory activities of tick saliva or components of the salivary gland in mammalian hosts or how these activities benefit tick-transmitted pathogens (Hovius et al., 2008; Pal and Fikrig, 2010). However, limited investigation addressed how vector immune responses influence the survival or persistence of specific pathogens within the tick. It is rather surprising that although ticks are known to encode components of a number of immune effector mechanisms, including humoral (classical AMPs) or cellular (phagocytosis) immune responses as well as evolutionary conserved signaling molecules or potentially active pathways (Toll, IMD, or JAK/STAT), their contribution in shaping *I. scapularis* immunity remains largely obscure. Tick-borne pathogens are evolved to persist and be transmitted by a specific tick species. Thus, it is conceivable that these pathogens coevolved and developed a successful and intimate relationship with the host. Additionally, to be successful in nature, these pathogens must have also evolved specific mechanisms to persist in the vector and evade innate immune insults. For example, when artificially challenged with the Lyme disease pathogen, *I. scapularis* ticks mount slower phagocytic responses and therefore, remain practically immunotolerant against spirochete infection (Johns et al., 2001). In contrast, another hard tick species, *D. variabilis*, when challenged with the same pathogen, generates a rapid and effective increase in phagocytic cells and clears the infection and thus, is highly immunocompetent against spirochete infection. With the availability of *I. scapularis* genome information and development of robust functional genomics and bioinformatics as well as the advent of efficient high-throughput genome sequencing tools, we expect exciting future research enhancing our knowledge of *I. scapularis* immunity and hope to address specific questions on the biology of tick immune responses against a diverse group of human pathogens. Together, these studies will contribute to a better understanding of the special biology of vector-microbe interaction and specific aspects of tick immunity and at the same time, contribute to the development of new strategies to combat pathogen transmission.

### Conflict of interest statement

The authors declare that the research was conducted in the absence of any commercial or financial relationships that could be construed as a potential conflict of interest.
